# Group A Streptococcal Pyomyositis in a Previously Healthy Six-year-old Girl

**DOI:** 10.7759/cureus.2168

**Published:** 2018-02-08

**Authors:** Daniel Tawfik, Wendy L Hobson

**Affiliations:** 1 Division of Pediatric Critical Care Medicine, Department of Pediatrics, Stanford University School of Medicine and Lucile Packard Children’s Hospital; 2 Department of Pediatrics, University of Utah School of Medicine

**Keywords:** hip pain, fever, pyomyositis, streptococcus

## Abstract

A six-year-old previously healthy girl was seen in an outpatient pediatric clinic in the western United States for thigh pain. The pain was accompanied by an initial fever and was most severe after periods of prolonged rest. During the evaluation, her exam rapidly progressed with severe episodic pain and tenderness of the right anteromedial thigh. Magnetic resonance imaging (MRI) demonstrated signal enhancement at the insertion of the right obturator externus muscle. Blood culture was positive for Group A Streptococcus. She was diagnosed with pyomyositis of the right obturator externus and was successfully treated with antimicrobials. This case demonstrates a rare case of streptococcal pyomyosits, in a temperate climate, without known predisposing factor or injury. We review the epidemiology of streptococcal pyomyositis in temperate climates and discuss the presentation of pyomyositis in children.

## Introduction

Pyomyositis is an uncommon condition in temperate climates, but its recognition has been increasing since the 1970’s. *Staphylococcus aureus* is the most common pathogen in temperate climate pyomyositis, but *Streptococcus pyogenes *has occasionally be identified, often in the setting of immune deficiency, recent varicella infection, or deep tissue trauma. We present a child with *Streptococcus pyogenes* pyomyositis in the absence of any known predisposing factors for this pathogen.

## Case presentation

A six-year-old previously healthy girl was seen in an academic general pediatric clinic in the western United States with a three-day history of right anteromedial thigh pain. The onset of pain was sudden, described as alternating between sharp and aching. On the first day of the pain, a temperature of 102 degrees Fahrenheit was noted at home, and acetaminophen was given causing resolution of fever and brief improvement in pain. Her pain was noted to be the worst when moving the leg after a prolonged period of sitting or lying down. She walked more slowly and had a limp. Headache and decreased energy were also noted on the first day of symptoms, but these did not continue. There were no other systemic symptoms and no pain in any other location. There were no recent illnesses. She did not have a sore throat, cough, congestion, or skin infections.

She was seen in the general pediatric clinic on the third day of symptoms, at which point she was afebrile. The physical exam showed an alert, pleasant, interactive young girl with mild tenderness over the right anteromedial thigh. She had an antalgic gait, but a full range of motion of the lower extremities with 2+ reflexes throughout. She had no tenderness over the right hip or knee. Tonsils were 2+ enlarged without exudate. However, on repeat examination 20 minutes later, her pain progressed to severe tenderness over the anteromedial right thigh. She had no other changes in the physical exam.

**Figure 1:**
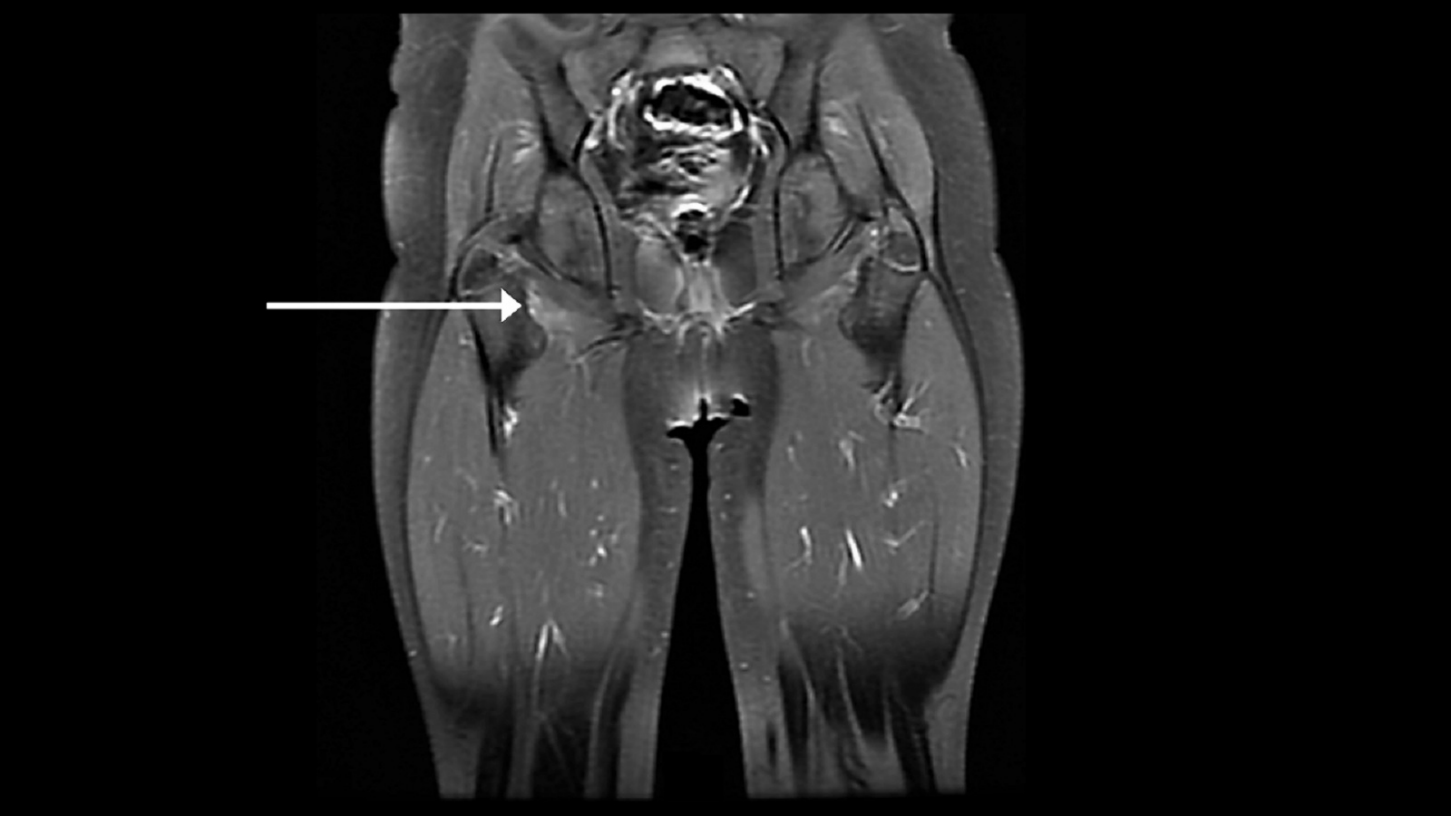
Subtle enhancement at the insertion of the right obturator externus muscle on T1 post-contrast magnetic resonance imaging

She was referred to the nearest pediatric hospital for magnetic resonance imaging (MRI), which showed subtly abnormal signal intensity and enhancement at the insertion of the right obturator externus muscle as shown in Figure 1, as well as small bilateral knee effusions with minimal synovitis. The white blood cell count was 14 K/uL with 10% band forms and 59% neutrophils. C-reactive protein was 4.6 mg/dL with an erythrocyte sedimentation rate of 45 mm/h. Creatine kinase was normal at 51 U/L. Blood culture was drawn.

The patient was admitted to the inpatient service and started on intravenous nafcillin 200 mg/kg/day and clindamycin 40 mg/kg/day for presumed pyomyositis. She was febrile to 39 degrees Celsius at the time of admission. The morning following admission, her thigh pain improved. Blood culture was positive for Group A Streptococcus species after 14 hours of incubation, and she was diagnosed with group A beta-hemolytic streptococcal pyomyositis of the right obturator externus muscle. Repeat blood culture showed no growth. Nafcillin was discontinued on hospital day two. She had no further thigh tenderness and was able to weight bear without difficulty on hospital day three. She was discharged on oral amoxicillin/clavulanate to complete a 21-day course. She recovered fully with no long-term sequelae.

## Discussion

Pyomyositis, a bacterial infection of the striated muscle typically with abscess formation, was first described by Scriba in 1885 and considered an endemic disease to tropical regions at that time [1]. It is an uncommon condition in temperate climates, where recent reports estimate it is responsible for one out of every 2000 to 4000 pediatric admissions in the United States [2], although recognition outside of the tropics has been increasing since the early 1970’s [3], primarily in children and young adults. The increase in incidence has been partially attributed to an increasing prevalence of immune deficiencies with a rise in diabetes mellitus and HIV/AIDS most prominent in the adult population, but many children with pyomyositis have no evidence of immunodeficiency [3].

Pyomyositis often results following an acute upper respiratory infection [4] or more severe infection such as meningitis, resulting in secondary bacteremia [3]. However, bacteremia alone is thought to be insufficient to result in pyomyositis, and it has been claimed that trauma to the muscle is required in order to develop the deep tissue infection. Yet a history of trauma is elicited in only 15%-50% of cases [3, 5]. The thigh was found to be the most common site involved in a large series of children with muscle abscesses, followed by the calf, buttock, and arm [6]. Our patient had a common location for pyomyositis but had an unusual presentation due to no identified antecedent infectious symptoms or history of trauma. Furthermore, pyomyositis often results from *Staphylococcus aureus* infection, responsible for 80%-90% of cases [5, 7]. *Streptococcus pyogenes*, as our patient had, is a much less common cause of pyomyositis, especially in immunocompetent individuals [3, 8] and in children [7]. Initial reports of *Streptococcus pyogenes* pyomyositis implicated a coincident primary varicella infection [9], but it has now been reported independent of varicella infection [10].

The clinical progression of pyomyositis typically begins with mild cramping, pain, and tenderness of the affected muscle, often with a low-grade fever. A physical exam may reveal localized induration of the affected muscle. Symptoms are typically mild enough that few patients present at this stage. The natural history of pyomyositis involves progression to an intermediate stage, characterized by increased muscle tenderness, fever, chills, anorexia, and overlying mild erythema of the skin. There is often abscess formation by this time. It appears that our patient was in the early portion of this stage upon presentation, as she did not have evidence of a clinically significant abscess within the muscle. Further progression without treatment results in the late stage, which is characterized by septic shock. Compartment syndrome with subsequent osteonecrosis as well as fatalities have been reported, making early diagnosis and treatment important for preventing morbidity and mortality [5].

## Conclusions

We present the case of a Group A beta-hemolytic streptococcal pyomyositis presenting with fever and hip pain in a previously healthy six-year-old girl. This case was unique in several ways. First, there was no history of predisposing antecedent illness which could be identified, and physical exam upon presentation was quite variable in severity, initially indicative of only very mild tenderness on initial examination. Second, this patient had no identifiable immunodeficiency or predisposing factors to the development of pyomyositis. Third, the causative organism, in this case, was found to be *Streptococcus pyogenes*, diagnosed via positive blood culture. Finally, there was no abscess formation evident on imaging, and the patient recovered fully without any procedural drainage, suggesting that the diagnosis was made fairly early in the clinical course, possibly earlier than many patients would present. Pediatricians should be aware of this disease in temperate climates, as it is being increasingly recognized, and early recognition and treatment are important for preventing septic shock and long-term sequelae.
